# Effects of a multi-component alcohol prevention program in the workplace on hazardous alcohol use among employees

**DOI:** 10.1186/s12889-023-16150-4

**Published:** 2023-07-24

**Authors:** Devy L. Elling, Ylva B. Almquist, Peter Wennberg, Kristina Sundqvist

**Affiliations:** 1 Department of Public Health Sciences, Stockholm University, Stockholm, SE-106 91 Sweden; 2 Department of Global Public Health, Karolinska Institutet, Stockholm, SE-171 77 Sweden; 3 Department of Psychology, Stockholm University, Stockholm, SE-106 91 Sweden

**Keywords:** Workplace intervention, Alcohol prevention, Hazardous alcohol use, Policy awareness

## Abstract

**Background:**

The workplace can be affected negatively by hazardous alcohol use, and intervening at an early stage remains a challenge. Recently, a multi-component alcohol prevention program, Alcohol Policy and Managers’ skills Training (hereafter, ‘APMaT’), was delivered at the organizational level. In a previous outcome evaluation, APMaT appeared to be effective at the managerial level. The current study takes a step further by aiming to evaluate the effectiveness of APMaT in decreasing the alcohol risk level among employees.

**Methods:**

Data from 853 employees (control: n = 586; intervention: n = 267) were gathered through a cluster-randomized study. To analyze changes in the odds of hazardous alcohol use among employees, multilevel logistic regression was applied using group (control vs. intervention), time (baseline vs. 12-month follow-up), and the multiplicative interaction term (group × time) as the main predictors. The intervention effect was further adjusted for sociodemographic characteristics and policy awareness.

**Results:**

No statistically significant difference was observed in the odds of hazardous alcohol use, although employees in the intervention group showed a larger decrease compared to the control group. This remained even after adjusting for several factors, including the sociodemographic factors and policy awareness.

**Conclusions:**

The findings are insufficient to determine the effectiveness of APMaT at the employee level at the current stage of the evaluation. Future studies should strive to identify issues with implementation processes in workplace-based alcohol interventions.

**Trial registration:**

The trial was retrospectively registered on 11/10/2019; ISCRTN ID: ISRCTN17250048.

**Supplementary Information:**

The online version contains supplementary material available at 10.1186/s12889-023-16150-4.

## Introduction

Hazardous alcohol use is associated with various health conditions, which in turn may lead to early labor exit [1, 2]. Since approximately 80% of the adult population in Sweden is employed, many individuals with hazardous alcohol use can be found in the workforce [3]. Accordingly, hazardous alcohol use can also impose problems at the organizational level. For instance, consequences stemming from alcohol use continue to be one of the main reasons employees miss work (absenteeism) [4], or are unable to work to their full capabilities (presenteeism) [5]. In addition to absenteeism and presenteeism, alcohol use in the workplace elevates the risk of injury [1], particularly for workers in occupations that involve high physical efforts [6]. Within the Swedish workplace context, employers are obliged to take appropriate actions when they identify individuals whose alcohol consumption could have a negative impact on the workplace [7]. In some sectors, such as the hospitality sector, alcohol is easily accessible and readily available because alcohol is often a part of their work routine [8]. Subsequently, this increases the susceptibility to hazardous use among employees [9, 10], and makes preventive intervention imperative. Despite the benefits of preventing hazardous alcohol use, early identification of hazardous alcohol use among employees remains a challenge.

A systematic review that evaluated the effectiveness of workplace-based alcohol interventions found mixed results [11]. Workplace interventions often include screening and monitoring [12, 13], brief interventions [14, 15], health promotion programs [16], and alcohol policies [12, 17]. The first two types of intervention often target hazardous use at the individual level [18], whereas the latter two types target the organization as a whole [12, 19]. Individual-level interventions (i.e. screening and monitoring and brief interventions) have mainly shown short-term effectiveness [20]. Although we might speculate on the long-term effectiveness of health promotion programs and alcohol policies [12, 21], the results are uncertain at this point. Moreover, many studies of workplace interventions have examined the effects of a single intervention component on alcohol use. Due to the complexity of the workplace as an arena for prevention programs, a multi-component intervention may be beneficial in reducing hazardous alcohol use among employees. A multi-component workplace intervention, that included an organizational alcohol policy and training of staff to increase the identification of hazardous consumers and provide the necessary skills to respond to alcohol-related issues, has shown promising results [19]. Assuming that an organizational alcohol policy is implemented properly by aligning expectations and understanding across organizational levels, it can be used as a tool to help managers act upon raised concerns regarding situations where employees’ alcohol use could negatively impact the workplace. The combination of policy implementation with health education can increase the likelihood of initiating an intervention at an early stage among employees with hazardous alcohol use, thereby preventing any potential negative consequences [22].

Recent studies have discussed the importance of co-creation with regard to interventions in the workplace [19, 23]. Having consistent engagement throughout the organizational hierarchies through co-creation can facilitate the development and implementation of an intervention [24]. Applying co-creation, for example to the case of forming an organizational alcohol policy, can result in successful implementation and dissemination in the long run, as it accounts for the organization’s needs [25], as well as workplace values and culture [26]. Similar to previous literature [19, 27], Alna, a division of the occupational health services that provides prevention services related to harmful use in workplaces [28], used the concept of co-creation in parts of a multi-component prevention program. The prevention program, henceforth ‘APMaT’ (Alcohol Policy and Managers’ skills Training), aims to prevent and reduce hazardous alcohol use and its consequences in the workplace. One of the intervention components – the development and implementation of an organizational alcohol policy – was planned to be co-created with human resources (HR) personnel and the organization’s management team. The details of the intervention components and their delivery are discussed in the methods section.

Our earlier study evaluated APMaT at the managerial level, focusing on managers’ inclination to initiate early alcohol intervention [29]. We found positive effects of APMaT on managers’ inclination, particularly in terms of their confidence in initiating a dialogue with employees about alcohol. Thus, APMaT appears to be effective in changing managers’ attitudes towards intervening (and possibly also their behavior). This study takes one step further in evaluating the intervention, by targeting hazardous alcohol use among the employees. While the positive change in managers’ inclination to initiate early alcohol intervention could very well affect employee behavior [30, 31], our focus will more exclusively be placed on processes occurring at the employee level. We hypothesize that APMaT, given an adequate implementation of the organizational alcohol policy [32], and the anticipated increase in policy awareness among employees, may have (indirect) effects on employees’ hazardous alcohol use. Accordingly, the aim of this study is to evaluate the effectiveness of a workplace alcohol prevention program at the employee level by examining changes in hazardous alcohol use among employees, and to understand whether employees’ policy awareness can explain any such changes.

## Materials and methods

### Study design

This study was a part of a larger evaluation project (Controlled Study of an Alcohol Preventive Interventions in Working Life; in Swedish: *Kontrollerad studie av AlkoholPReventiva Insatser i arbetslivet*—KAPRI), aimed at evaluating the effectiveness of an alcohol preventive intervention in the workplace.

This study was a two-armed cluster randomized controlled study with parallel groups. A cluster-randomized trial was chosen because the prevention program was delivered at the organizational level, with a special focus on workplace policies and managers’ skills at identifying hazardous alcohol use at an early stage. The program was designed and delivered by Alna, a division of an occupational health service organization that provides prevention services related to harmful use (e.g. drugs, gambling) to workplaces [28].

### Intervention

The intervention had two components, of which the first was based on co-creation. During this component, HR personnel and the management team within each organization improved their organization’s alcohol policy, and thereafter drafted an implementation plan together with Alna.

In terms of the second component, managers (including, e.g. supervisors and team leaders) completed a two-part workshop with Alna, conducted in one day or two half-days. The first part aimed to increase managers’ knowledge regarding alcohol use in the workplace and its consequences, whereas the second part aimed to improve managers’ skills in identifying the early signs of hazardous alcohol use, and increase managers’ confidence to have difficult discussions with their employees. A detailed description of the intervention can be found in the study protocol [33].

Both components of APMaT were delivered at the organizational level, where the program was assumed to have been implemented to some degree within the current study period. Therefore, this study was operationalized under the assumption that both intervention components have had some effects on the outcome measures at the employee level.

### Recruitment of organizations and participants

In this study, the organizations were recruited by screening Alna’s company register prior to recruitment. Based on Alna’s company register, representatives of organizations with at least one hundred employees were contacted by e-mail and telephone, sharing the project’s rationale. Previous research has shown that certain employment sectors (e.g. transport, construction, and hospitality) tend to have an overrepresentation of alcohol use among their employees [34], and thus were prioritized during the recruitment process.

According to the KAPRI project design, participants were considered eligible if they were employed at the recruited organizations at both the baseline and 12-month follow-up measurement time points.

### Randomization and blinding

Randomization was performed at the organizational level. The thirteen participating organizations were grouped into clusters of two to four organizations based on their sector (e.g. hospitality) and size (medium-sized or large). Next, each cluster was randomized, and organizations were allocated to either the control or the intervention group using an online web service (random.org). For example, two large organizations within the hospitality sector were grouped, and they were randomly allocated to either the control or intervention group. Organizations in the intervention group received the intervention soon after baseline data were collected, and organizations in the control group were put on a waitlist and asked to continue with their usual practice. The organizations in the control group received the same intervention after 12-month follow-up data have been collected.

It was not possible to blind the organizations or the managers due to the design of the prevention program. Additionally, two organizations dropped out after randomization because they were not satisfied with their group allocation, leaving a total of 11 organizations (control: n = 5 organizations; intervention: n = 6 organizations) participating in the larger evaluation at baseline.

The prevention program was delivered by consultants from Alna. Their roles were to advise and act as health educators. None of the consultants had any role in the program evaluation.

### Data collection

Data were gathered at baseline (August–October 2018) and follow-up (August–October 2019) through a self-administered online survey. The survey included questions about sociodemographic characteristics (sex, age, and educational level), awareness of organizational alcohol policy, and alcohol use.

A link to the survey was distributed through e-mail or, if this option was unavailable, a general link on the organization’s internal website. To increase the response rate, three reminder e-mails were sent at one-week intervals to participants who had not completed the survey, and two reminders were sent at one-week intervals via the organization’s representative and the organization’s internal website for participants who received a general link.

### Measures

The primary outcome was *hazardous alcohol use*. This was measured by the Alcohol Use Disorder Identification Test (AUDIT). The AUDIT is a validated screening tool for alcohol use developed by the World Health Organization (WHO) [35]. Alcohol use was measured by calculating the total scores for the AUDIT [35]. The total AUDIT scores were then dichotomized based on the Swedish recommendation into abstention and low-risk alcohol use (female: < 6 points; males: < 8 points), or hazardous alcohol use (females: ≥ 6 points; males: ≥ 8 points) [36].

*Policy awareness* was used as a potential explanation for any changes identified in alcohol use among employees. This was measured using the following three items from the survey: ‘If I have a problem with alcohol, I think that my workplace would be able to help me’ (*supportive organization to oneself*), ‘If a colleague has a problem with alcohol, I think that my workplace would be able to help them’ (*supportive organization to colleagues*), and ‘How well do you know your workplace’s alcohol policy?’ (*alcohol policy knowledge*). These items were measured on a 5-point Likert scale, ranging from 1 (strongly disagree) to 5 (strongly agree). Because all three items measured awareness of alcohol policy from different perspectives, they were measured separately.

The variables used to describe the study population and examine differences between the control and intervention groups at baseline were *sex* (male, female), *age group* (16–24, 25–34, 35–44, 45–54, 55–64, ≥ 65 years), and *education level* (primary, upper secondary, tertiary). Due to low cell counts, some age groups were collapsed (≤ 34, 35–44, 45–54, ≥ 55 years).

### Sample size

The sample size in this study was dictated by the sample size estimation of the KAPRI project, determined at the organizational level based on the expected number of individuals within each of the organizations. The assumptions for sample size estimation for the larger evaluation project have previously been described in a study protocol [33].

### Statistical analyses

In order to describe the study population and examine the differences between the control and intervention groups, the frequency and percentage of hazardous alcohol use among employees were calculated using Pearson’s chi-square test. The mean value and standard deviation for alcohol use and policy awareness were calculated using independent t-test. The differences between employees in the control and intervention groups regarding the sociodemographic variables were only observed at baseline.

Because a large proportion of data was missing due to loss to follow-up, and the outcome measure was based on two-time points, imputation of missing values was considered problematic. Thus, all analyses followed a complete case analysis approach. Logistic regression analyses were performed to assess hazardous alcohol use among employees, with abstainers and individuals with low-risk alcohol use serving as the reference group. Since employees were clustered within the organizations, multilevel modeling was applied to account for both fixed and random effects. Moreover, a multiplicative interaction term of *group* (control vs. intervention) and *time* (baseline vs. follow-up) was created to observe changes in hazardous alcohol use.

First, changes in hazardous alcohol use were estimated using group (control vs. intervention), time (baseline vs. follow-up), and the multiplicative interaction term (group × time) as the main predictors (Model 1). Next, this model was adjusted for sex, age, and education level (Model 2). Finally, this model was adjusted for awareness of alcohol policy (Model 3) to explore any potential mediating effect. In all the adjusted models, the inclusion of covariates accounted for changes in those covariates between baseline to follow-up. All results were presented as odds ratios (OR) with 95% confidence intervals (CI). An alpha level of p < 0.05 was considered statistically significant.

#### Additional analyses

Several additional analyses were conducted. First, employee characteristics among those who had complete information for the baseline questionnaire were compared with those who had complete information for the follow-up, in order to examine any systematic differences in loss to follow-up (Table S1). Second, by entering each of the measures as a moderator separately, we examined whether there were any moderation effects of policy awareness (Table S2). Third, we re-examined our primary analysis stratified by sex and age to see if any potential differences of the intervention effects could be observed (Tables S3 and S4). Fourth, to examine whether the dichotomization of AUDIT score could bias the estimates, Model 1 was re-examined using the total AUDIT score as a linear outcome (Table S5). Here, we also examined whether a larger sample size, based on the number of employees who had complete information for the baseline questionnaire (n = 2,248), would differ significantly from the primary analyses. Using a maximum likelihood approach, we estimated whether attrition could have impacted the effects of the intervention on the outcome measures with a dichotomized AUDIT score (Table S6), as well as with total AUDIT scores as a linear outcome (Table S7). Furthermore, to examine whether there were any potential intervention effects on policy awareness, Model 1 was re-examined (Table S8).

All analyses were performed using Stata Statistical Software version 16 (StataCorp, College Station, TX, USA).

### Ethical considerations

The Ethical Review Board of Stockholm granted ethical approval (dnr 2018/634 − 31/5). The KAPRI project was retrospectively registered on 11/10/2019 in the ISCRTN register (ISCRTN.com: ISRCTN17250048). Informed consent was obtained from all subjects involved in the study. Representatives in each organization circulated information about the project via their respective internal websites, after which the survey was distributed to all employees. Information about the study and a statement that implied participants’ consent was presented at the top of the survey. Therefore, responding to the survey was considered as consent to the study.

## Results

### Study population

#### Recruitment of organizations and retention

**Fig. 1:**
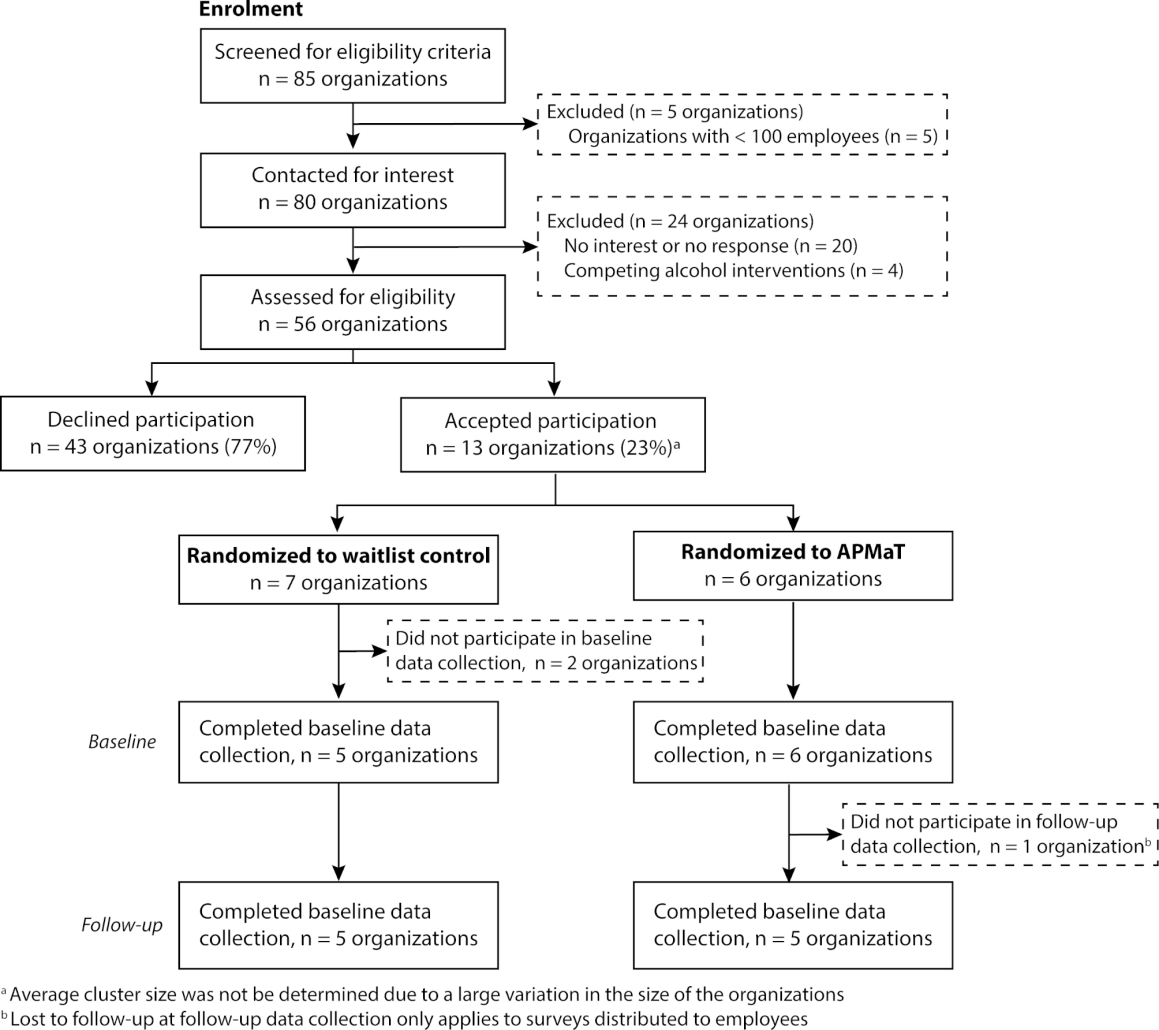
Schematic overview of organizations from recruitment to follow-up in the cluster-randomized controlled study

A total of 56 eligible organizations were invited, and 13 organizations (23%) initially agreed to participate in KAPRI. After randomization, two organizations withdrew from the study because they were dissatisfied with group allocation, leaving a total of 11 organizations (control: n = 5 organizations; intervention: n = 6 organizations) participating in KAPRI at baseline. At follow-up, all the employees of one organization were lost to follow-up, resulting in a total of five organizations in both the control and intervention groups (Fig. 1).

#### Recruitment of participants and retention

**Fig. 2:**
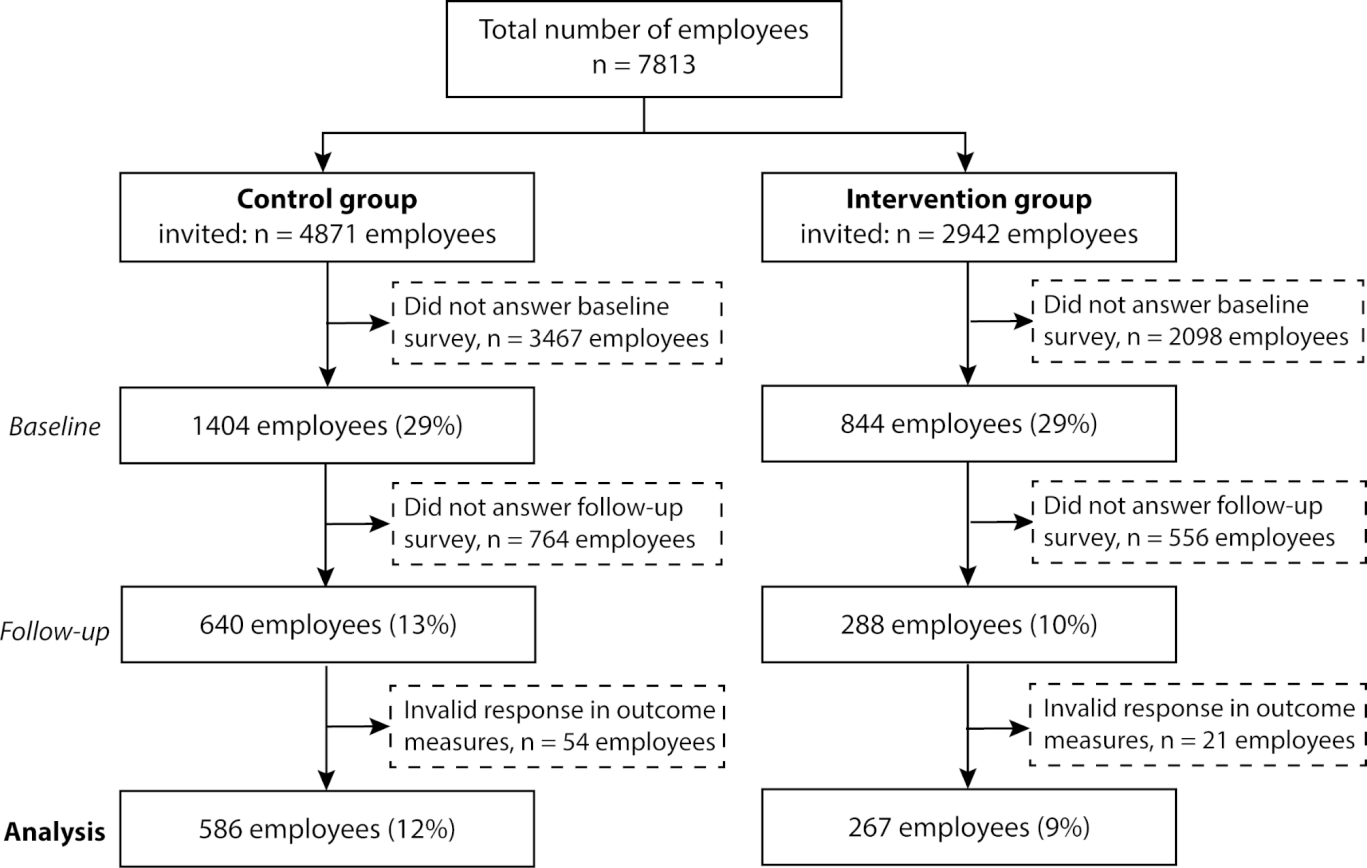
Schematic overview of participants from recruitment to follow-up in the cluster-randomized controlled study

A total of 7813 employees were invited to partake in the survey at baseline, of which 2,248 employees completed the baseline survey (control: n = 1,404 employees; intervention: n = 844 employees). Among all employees that were initially invited to participate at baseline, 640 employees (13%) from the control group and 288 employees (10%) in the intervention group participated in the follow-up survey (Fig. 2). The characteristics of employees recruited at baseline and retained at follow-up are presented in the supplementary materials (Table S1).

In the current study, the analytical sample included employees who responded to both the baseline and follow-up surveys, and did not have any invalid responses to the outcome measures. This resulted in a total of 853 participants (control: n = 586 employees; intervention: n = 267 employees).

Table 1 shows the sociodemographic characteristics of the analytical sample at baseline. The majority of employees were male in both the control (55.3%) and the intervention groups (50.9%), and most of the employees were under 55 years of age. While most of the employees in the control group had upper secondary education (51.0%), employees in the intervention group had tertiary education (49.4%). Although some differences in sociodemographic characteristics among employees were observed at baseline, these were not statistically significant.

**Table 1:** Sociodemographic characteristics of the analytical sample at baseline (n = 853)

Variable, n (%)	Control (n = 586)	Intervention (n = 267)	p
Sex			
Male	324 (55.3)	136 (50.9)	0.237
Female	262 (44.7)	131 (49.1)	
Age group			
≤ 34 years	145 (24.7)	64 (24.0)	0.302
35–44 years	167 (28.5)	92 (34.5)	
45–54 years	161 (27.5)	69 (25.8)	
≥ 55 years	113 (19.3)	42 (15.7)	
Education level ^a^			
Primary education	28 (4.8)	12 (4.5)	0.370
Upper secondary education	297 (51.0)	123 (46.1)	
Tertiary education	258 (44.3)	132 (49.4)	

^a^ Missing value due to internal missing values of a few individuals

Differences between the control and the intervention group were calculated using Pearson’s chi-square test

Table 2 presents differences between employees in the control and intervention groups at the two-time points. At baseline, the mean AUDIT score was higher in the intervention group (4.30 points) compared to the control group (4.17 points). A slight decrease in the mean AUDIT score was observed for both groups at follow-up, although a larger decrease was observed among employees in the intervention group. The majority of employees in both groups were classified as abstainers or had a low-risk use at baseline (control: 82.6%; intervention: 81.3%). At follow-up, a slight increase in the proportion of employees who were abstainers or had low-risk alcohol use was observed in the control group (82.8%), and a larger increase in the same measure was observed in the intervention group (84.3%). Differences in policy awareness between employees in the control and intervention groups were observed, particularly regarding organizational support for employees and their colleagues. These differences were statistically significant and remained at follow-up. While the level of alcohol policy knowledge differed statistically between employees in each group at baseline, the difference was not observable at follow-up. No differences were found between employees with partial and complete attrition in the outcome measures (Table S1).

**Table 2:** Descriptive statistics of the analytical sample (n = 853)

Variable	Baseline			Follow-up		
	Control (n = 586)	Intervention (n = 267)	p	Control (n = 586)	Intervention (n = 267)	p
Alcohol use^a^, mean (s.d.)	4.17 (3.4)	4.30 (3.2)	0.617	4.13 (3.5)	4.12 (3.0)	0.964
Alcohol use^b^, n (%)						
Abstention or low-risk use	484 (82.6)	217 (81.3)	0.640	485 (82.8)	225 (84.3)	0.585
Hazardous use	102 (17.4)	50 (18.7)		101 (17.2)	42 (15.7)	
Policy awareness^a^, mean (s.d.)						
Supportive organisation to oneself	3.48 (1.2)	3.86 (1.0)	**≤ 0.001**	3.54 (1.1)	3.97 (1.0)	**≤ 0.001**
Supportive organisation to colleagues	3.47 (1.6)	3.85 (1.0)	**≤ 0.001**	3.52 (1.1)	3.94 (1.0)	**≤ 0.001**
Alcohol policy knowledge	2.99 (1.5)	3.33 (1.3)	**≤ 0.001**	3.35 (1.4)	3.48 (1.3)	0.203

s.d.: standard deviation. Bold font: p < 0.05

^a^ Differences between the control and intervention groups were calculated with independent t-test

^b^ Differences between the control and intervention groups were calculated with Pearson’s chi-square test

### Effectiveness of the intervention

Table 3 presents the effectiveness of APMaT on hazardous alcohol use among employees. Model 1 shows no significant decrease in hazardous use among employees in the intervention group compared to the control group at follow-up (OR: 0.82; 95% CI: 0.48 to 1.41). This finding remains the same in Model 2, which adjusts for sociodemographic characteristics (OR: 0.82; 95% CI: 0.47 to 1.42). Corresponding to the study aim, Model 3 adjusted for policy awareness in order to explore potential mediation. This did not lead to any changes in the overall results (OR: 0.82; 95% CI: 0.47 to 1.42).

**Table 3:** Intervention effect on employees’ hazardous alcohol use measured using the Alcohol Use Disorder Identification Test (AUDIT) scores in the analytical sample (n = 853). Results from multilevel logistic regression

	Hazardous alcohol use (Ref: Abstention or low-risk use)					
	Model 1^a^		Model 2^b^		Model 3^c^	
	OR	95% CI	OR	95% CI	OR	95% CI
Group, control group = ref.	1.09	0.75, 1.59	1.07	0.73, 1.57	1.14	0.77, 1.67
Time, baseline = ref.	0.99	0.73, 1.34	1.02	0.75, 1.38	1.03	0.76, 1.41
Interaction (group×time)	0.82	0.48, 1.41	0.82	0.47, 1.42	0.82	0.47, 1.42

OR: Odds Ratio; CI: Confidence Interval

^a^ Mutually adjusted for group (control vs. intervention group), time (baseline vs. 12-month follow-up), and the interaction term (group × time)

^b^ Mutually adjusted for group (control vs. intervention group), time (baseline vs. 12-month follow-up), the interaction term (group × time), sex, age, and educational attainment

^c^ Mutually adjusted for group (control vs. intervention group), time (baseline vs. 12-month follow-up), the interaction term (group × time), sex, age, educational attainment, and policy awareness among employees

## Discussion

This study evaluated the effectiveness of APMaT, a workplace-based alcohol prevention program, by examining changes in hazardous alcohol use among employees. A larger decrease in hazardous alcohol use among employees in the intervention group was detected. However, this difference was not statistically significant. Similar results were observed after adjusting the association for sociodemographic characteristics, in line with previous literature [19, 37–39]. While we initially identified policy awareness among employees as a potential mediator, our study did not provide any support for this. Given the current findings, the effectiveness of APMaT in decreasing hazardous alcohol use among employees remains uncertain.

In our previous evaluation of APMaT, we found effects of the intervention on managers’ inclination to intervene [29]. It is reasonable to expect that this increase in inclination would improve the implementation of the organizational alcohol policy, which includes an alignment regarding the perception of organizational values and the contents of the policy [40]. Consequently, adequate policy implementation can increase policy awareness among employees. One of our additional analyses (Table S8, supplementary materials) was largely in line with this notion: indeed, here we found APMaT to be associated with an overall positive change in policy awareness. Theoretically, increasing employees’ knowledge can lead to a positive change in their attitudes, which subsequently may result in a modification of behavior [19, 23, 38]. The fact that we could not provide any empirical support for APMaT having effects on hazardous alcohol use could be due to several reasons. Firstly, alcohol use is multifaceted (e.g. drinking outside of the workplace [10], and ambiguous boundaries between working hours and leisure time [41]). Managers might, despite the intervention being effective in improving their *inclination* to intervene, still lack the knowledge and practical skills needed to identify and intervene in response to employees with hazardous alcohol use [37]. Second, it should be noted that the intervention did not affect all three indicators of policy awareness in the same way. While leading to positive changes in the employees’ assessment of their organization being supportive if they or their colleagues would have problems with alcohol, a negative effect was found for knowledge about the organization’s actual alcohol policy. The organizational alcohol policy was devised by HR personnel and the management team, and managers were expected to distribute the organizational policy document, as well as to explain what constitutes hazardous alcohol use and the consequences for non-compliance [40]. This could be exemplified by actions that will be taken in case of violation of rules regarding hazardous use [8]. If the alignment through all organizational levels was not reached, it is possible that lower-level employees had a different or more limited understanding of the (recent changes in) alcohol policy. In turn, this might have reduced the effectiveness of the intervention on hazardous alcohol use.

In order to identify possible reasons for the non-significant findings, we conducted a series of additional analyses. We did not detect any major differences in our additional analyses (presented in the supplementary materials) compared to our primary analyses; however, some insights into the factors that could have influenced our findings arose. Firstly, we could not detect any moderation effect of policy awareness on changes in hazardous alcohol use (Table S2). This made it difficult to understand whether and/or how the intervention was implemented at the highest level of the organizations (i.e. managerial level). Here, we can only speculate that our estimates were due to insufficient program implementation rather than other contextual factors, such as being informed of the workplace participation in KAPRI, or previous experiences regarding alcohol-related issues in the workplace. Such factors, although beyond the scope of the current study, could have influenced our findings. For instance, some organizations may be more engaged in APMaT because of their previous experiences of alcohol-related issues in the workplace. The engagement towards the intervention could therefore influence employees’ attitudes towards alcohol, and possibly their behaviors, which could consequently, bias the intervention’s effects on alcohol use.

Secondly, our stratified analyses by sex (Table S3) and age (Table S4) did not show any statistically significant differences in terms of the intervention effects on hazardous alcohol use. This was somewhat surprising, given that findings from previous literature showed that females tend to have lower alcohol use compared to their male peers [42], and younger employees are more likely to engage in hazardous alcohol use than older employees [4]. It should, however, be noted that the non-statistical significance in our findings reflects on the impact of APMaT on alcohol use, and not on the patterns of hazardous alcohol use in the general working population.

Thirdly, the intervention effect on alcohol use was unlikely to be influenced by the dichotomization of the total AUDIT score, as the estimates of alcohol use as a linear outcome did not change the overall findings of this study (Table S5). The skewed distribution of the total AUDIT scores in our initial analysis determined that setting the cut-off of abstention/low-risk and hazardous alcohol use based on the Swedish recommendation would provide a better overview regarding the importance of early interventions within the Swedish workplace context.

Fourthly, the large loss to follow-up could be attributed to some difficulties when distributing the survey. We found that the survey that was distributed via general link through the organization’s internal website had the highest attrition compared to the survey that was distributed through e-mail. We re-examined our primary analyses; however, we could not detect any significant differences in the intervention effects on alcohol use with a larger sample size (Tables S6 and S7).

Some strengths and limitations of the current study were identified. First, hazardous alcohol use was measured using a scale validated by the WHO, making the results of this study comparable to other research on the topic. Second, randomization ensured that the differences between the control and intervention groups at the organizational level were small, which minimized the risk of selection bias [43]. Third, the follow-up period was longer than in many other studies examining the effectiveness of workplace-based prevention programs [19, 37–39]. Finally, this study applied multilevel modeling which, unlike approaches using pooled estimates of the control and intervention groups, accounts for organizational differences.

This study was limited by the distal outcome of interest to the intervention components, particularly within the current follow-up time. The one-year follow-up period constitutes quite a sparse grid that might miss capturing short-term changes. It is possible that hazardous alcohol use was not an ideal outcome since the implied level of severity could require more comprehensive types of interventions (spanning more domains than work-life) and changes might take a longer time. Moreover, the survey design may have influenced the findings. The self-administered survey may lead to social desirability bias since employees may underestimate their alcohol use or overestimate their policy awareness. It is possible that responding to the survey in and of itself led to increased awareness among respondents regarding their alcohol use rather than their policy awareness. In addition, it was not possible to distinguish hazardous alcohol use within and beyond the workplace due to data availability. The data regarding other potential indicators of alcohol use to understand employees’ attitudes towards alcohol, experiences of alcohol-related harms, and general workplace culture surrounding alcohol was not available—something that could explain current study findings in understanding the influence of factors outside of the workplace on alcohol use. Finally, one of the major limitations was the fact that the study population was smaller than originally planned largely due to a low response rate at baseline and loss to follow-up, which limited the ability to detect the previously hypothesized effect size. In the current study, a post-hoc power analysis of the crude model showed that a sample size of at least 1,255 employees would have been required to achieve 80% power at an alpha level of 0.05, something that could not be achieved due to the loss of follow-up and internal missing values of the outcome measures. Since the power of the current study was lower than expected, this could explain the study findings. The discrepancy between the total number of participants between the control and intervention groups was largely due to organizational dropout after randomization and a large proportion of loss to follow-up. As such, this may bias the intervention effect, resulting in a decrease in the generalizability of the current findings. Nevertheless, in the additional analyses, where the larger sample size was obtained using the maximum likelihood approach, the overall conclusions did not change.

## Conclusions

The findings could not provide any empirical evidence to support the effectiveness of the studied intervention, APMaT, in changing alcohol use among employees within the current observation time. The effectiveness of APMaT might therefore be promising with a lengthier follow-up period. However, considering the complexity of delivering a prevention program in a dynamic workplace context, processes in the implementation of the program should be explored further. Despite the results in the present study, APMaT might be effective in other contexts if different delivery and implementation strategies were employed, or with a lengthier follow-up. While co-creation is the ambition of APMaT, employees were not engaged in the co-creation of the organizational alcohol policy or its enforcement. Prospective workplace prevention programs with similar objectives should increase the involvement of other actors within the organization beyond the management team while considering the organizational norms and culture in the early stages of co-creation.

## Electronic supplementary material

Below is the link to the electronic supplementary material.

Supplementary Material 1

## Data Availability

The datasets generated and/or analyzed during the current study are not publicly available due to sensitive personal information but may be available from the principal investigators, Professor Peter Wennberg (peter.wennberg@su.se) or Dr. Kristina Sundqvist (kristina.sundqvist@su.se) at reasonable request.

## List of abbreviations

APMaT: Alcohol Policy and Managers’ skills Training
AUDIT: Alcohol Use Disorder Identification Test
CI: Confidence intervals
HR: Human resources
KAPRI: Controlled Study of an Alcohol Preventive Interventions in Working Life; in Swedish:*Kontrollerad studie av AlkoholPReventiva Insatser i arbetslivet*
OR: Odds ratios
WHO: World Health Organization
