# Supplementary material for: Effects of a multi-component alcohol prevention program in the workplace on hazardous alcohol use among employees
Source: BMC Public Health. 2023 Jul 24;23:1420. doi: 10.1186/s12889-023-16150-4 (PMC10367231; doi:10.1186/s12889-023-16150-4)
Supplement: Supplementary file 1 — Supplementary Material 1 [file 12889_2023_16150_MOESM1_ESM.docx]

# Supplementary materials

**Table S1.** Characteristics of employees with complete information at baseline (N = 2,248) and employees with complete information at follow-up (n = 928), by group allocation.

|  | Baseline (N = 2,248) | | | Follow-up^b^ (n = 928) | | | p^c^ |
| --- | --- | --- | --- | --- | --- | --- | --- |
| **n (%)** ^a,d^ | **Control**  **(n = 1,404)** | **Intervention**  **(n = 844)** | **p** | **Control**  **(n = 640)** | **Intervention**  **(n = 288)** | **p** |  |
| Sex |  |  |  |  |  |  |  |
| Male | 774 (55.1) | 390 (46.2) | **≤0.001** | 353 (55.3) | 143 (40.3) | 0.160 | 0.009 |
| Female | 630 (44.9) | 454 (53.8) |  | 284 (44.7) | 141 (49.7) |  |  |
| Age group |  |  |  |  |  |  |  |
| ≤ 34 years | 480 (34.7) | 268 (31.9) | 0.373 | 158 (24.7) | 67 (23.3) | 0.356 | 0.079 |
| 35–44 years | 365 (26.4) | 232 (27.7) |  | 172 (26.8) | 90 (31.3) |  |  |
| 45–54 years | 311 (22.5) | 209 (24.9) |  | 168 (26.3) | 79 (27.4) |  |  |
| ≥ 55 years | 228 (16.5) | 130 (15.5) |  | 142 (22.2) | 52 (18.1) |  |  |
| Alcohol use ^e^, mean (s.d.) | 4.2 (3.2) | 3.7 (3.2) | **≤0.001** | 4.2 (3.7) | 4.0 (3.3) | 0.350 | 0.076 |
| Alcohol use |  |  |  |  |  |  |  |
| Abstention or low-risk use | 1103 (82.6) | 665 (84.7) | 0.211 | 528 (85.0) | 236 (86.1) | 0.666 | 0.629 |
| Hazardous use | 232 (17.4) | 120 (15.3) |  | 93 (15.0) | 38 (13.9) |  |  |
| Policy awareness ^e^, mean (s.d.) |  |  |  |  |  |  |  |
| Supportive organization to oneself | 2.8 (1.2) | 3.1 (1.1) | **≤0.001** | 2.8 (1.1) | 3.1 (0.9) | **≤0.001** | 0.766 |
| *Table S1. continued* |  |  |  |  |  |  |  |
| Supportive organization to colleagues | 2.7 (1.2) | 2.8 (1.3) | 0.371 | 2.8 (1.1) | 3.1 (0.9) | **≤0.001** | **≤0.001** |
| Alcohol policy knowledge | 3.1 (1.4) | 3.2 (1.3) | 0.732 | 3.1 (1.2) | 3.0 (1.1) | 0.221 | 0.460 |
| s.d.: standard deviation. Bold font: p < 0.05  ^a^ Missing value due to internal missing values of a few individuals.  ^b^ Responded to both baseline and follow-up surveys.  ^c^ Differences between characteristic of participants in the control and intervention groups at baseline and follow-up survey, calculated with Pearson’s chi-square test for categorical variables and independent t-test for continuous variables.  ^d^ Differences between the control and intervention groups were calculated with Pearson’s chi-square test.  ^e^ Differences between the control and intervention groups were calculated with independent t-test. | | | | | | | |

**Table S2**. Intervention effect on employees’ hazardous alcohol use measured using the Alcohol Use Disorder Identification Test (AUDIT) scores moderated by policy awareness in the analytical sample (n=853). Results from multilevel logistic regression.

|  | Hazardous alcohol use (Ref: Abstention or low-risk use) | | | | | |
| --- | --- | --- | --- | --- | --- | --- |
|  | **OR^a^** | **95% CI** | **OR^b^** | **95% CI** | **OR^c^** | **95% CI** |
| Group, control group=ref. | 0.86 | 0.24, 3.10 | 0.89 | 0.24, 3.27 | 1.50 | 0.59, 3.83 |
| Time, baseline=ref. | 0.60 | 0.24, 1.49 | 0.80 | 0.32, 2.01 | 1.13 | 0.55, 2.35 |
| Interaction (group×time) | 1.51 | 0.22, 10.35 | 1.25 | 0.18, 8.55 | 0.77 | 0.19, 3.14 |
| Moderation effect | 0.84 | 0.51, 1.38 | 0.90 | 0.54, 1.47 | 1.02 | 0.68, 1.53 |
| CI: Confidence Interval; OR: Odds Ratio  ^a^ Mutually adjusted for group (control vs intervention group), time (baseline vs 12-month follow-up), the interaction term (group × time), and the moderator (group × time × supportive organization to oneself).  ^b^ Mutually adjusted for group (control vs intervention group), time (baseline vs 12-month follow-up), the interaction term (group × time), and the moderator (group × time × supportive organization to colleagues).  ^c^ Mutually adjusted for group (control vs intervention group), time (baseline vs 12-month follow-up), the interaction term (group × time), and the moderator (group × time × alcohol policy knowledge). | | | | | | |

**Table S3**. Intervention effect on employees’ hazardous alcohol use measured using the Alcohol Use Disorder Identification Test (AUDIT) scores in the analytical sample (n=853), stratified by sex. Results from multilevel logistic regression.

|  | | | | | | | | |
| --- | --- | --- | --- | --- | --- | --- | --- | --- |
|  | **Hazardous alcohol use (Ref: Abstention or low-risk use)** | | | | | | | |
|  | **Male** | | | | **Female** | | | |
|  | **OR^a^** | **95% CI** | **OR^b^** | **95% CI** | **OR^a^** | **95% CI** | **OR^b^** | **95% CI** |
| Group, control group=ref. | 1.35 | 0.81, 2.26 | 1.59 | 0.93, 2.71 | 0.86 | 0.49, 1.49 | 0.77 | 0.43, 1.36 |
| Time, baseline=ref. | 1.03 | 0.68, 1.56 | 1.10 | 0.72, 1.68 | 0.95 | 0.61, 1.47 | 0.97 | 0.61, 1.53 |
| Interaction (group × time) | 0.85 | 0.41, 1.76 | 0.82 | 0.39, 1.74 | 0.78 | 0.35, 1.76 | 0.81 | 0.35, 1.86 |
| CI: Confidence Interval; OR: Odds ratios.  ^a^ Mutually adjusted for group (control vs intervention group), time (baseline vs 12-months follow-up), and the interaction term (group × time).  ^b^ Mutually adjusted for group (control vs intervention group), time (baseline vs 12-months follow-up), the interaction term (group × time), age, educational attainment, and policy awareness among employees. | | | | | | | | |

**Table S4**. Intervention effect on employees’ hazardous alcohol use measured using the Alcohol Use Disorder Identification Test (AUDIT) scores in the analytical sample (n=853), stratified by age group. Results from multilevel logistic regression.

|  | | | | | | | | |
| --- | --- | --- | --- | --- | --- | --- | --- | --- |
|  | **Hazardous alcohol use (Ref: Abstention or low-risk use)** | | | | | | | |
|  | **≤ 34 years** | | **35–44 years** | | **45–54 years** | | **≥ 55 years** | |
|  | **OR^a^** | **95% CI** | **OR^a^** | **95% CI** | **OR^a^** | **95% CI** | **OR^a^** | **95% CI** |
| Group, control group=ref. | **2.04** | **1.08, 3.85** | 0.85 | 0.43, 1.68 | 0.54 | 0.22, 1.29 | 1.08 | 0.32, 3.66 |
| Time, baseline=ref. | 0.93 | 0.53, 1.63 | 1.25 | 0.73, 2.15 | 0.95 | 0.53, 1.70 | 0.79 | 0.31, 2.01 |
| Interaction (group×time) | 1.20 | 0.48, 3.00 | 0.50 | 0.18, 1.40 | 0.80 | 0.22, 2.88 | 0.81 | 0.13, 5.00 |
| CI: Confidence Interval; OR: Odds ratios.  Bold font: p < 0.05  ^a^ Mutually adjusted for group (control vs intervention group), time (baseline vs 12-months follow-up), and the interaction term (group × time). | | | | | | | | |

**Table S5.** Intervention effect on alcohol use measured using the Alcohol Use Disorder Identification Test (AUDIT) scores as a linear outcome among employees in the analytical sample (n=853). Results from linear mixed model.

|  | Alcohol consumption | | | | | |
| --- | --- | --- | --- | --- | --- | --- |
|  | **Beta^a^** | **95% CI** | **Beta^b^** | **95% CI** | **Beta^c^** | **95% CI** |
| Group, control group=ref. | 0.12 | -0.36, 0.61 | 0.15 | -0.33, 0.62 | 0.22 | -0.25, 0.69 |
| Time, baseline=ref. | -0.04 | -0.42, 0.35 | -0.00 | -0.38, 0.37 | 0.01 | -0.36, 0.38 |
| Interaction (group×time) | -0.13 | -0.82, 0.55 | -0.13 | -0.79, 0.53 | -0.13 | -0.79, 0.54 |
| CI: Confidence Interval  ^a^ Mutually adjusted for group (control vs. intervention group), time (baseline vs. 12-months follow-up), and the interaction term (group × time)  ^b^ Mutually adjusted for group (control vs. intervention group), time (baseline vs. 12-months follow-up), the interaction term (group × time), sex, age, and educational attainment  ^c^ Mutually adjusted for group (control vs. intervention group), time (baseline vs. 12-months follow-up), the interaction term (group × time), sex, age, educational attainment, and policy awareness among employees | | | | | | |

**Table S6.** Intervention effect on hazardous alcohol use measured using the Alcohol Use Disorder Identification Test (AUDIT) scores among employees with complete outcome information at baseline (n=2,228). Results from multilevel logistic regression.

|  | Hazardous alcohol use (Ref: Abstention or low-risk use) | | | | | |
| --- | --- | --- | --- | --- | --- | --- |
|  | **Model 1^a^** | | **Model 2^b^** | | **Model 3^c^** | |
|  | **OR** | **95% CI** | **OR** | **95% CI** | **OR** | **95% CI** |
| Group, control group=ref. | 0.83 | 0.66, 1.06 | 0.86 | 0.67, 1.11 | 1.02 | 0.78, 1.32 |
| Time, baseline=ref. | 0.85 | 0.65, 1.11 | 0.94 | 0.71, 1.23 | 0.92 | 0.70, 1.23 |
| Interaction (group×time) | 1.08 | 0.67, 1.75 | 1.04 | 0.63, 1.71 | 0.91 | 0.55, 1.50 |
| CI: Confidence Interval; OR: Odds Ratio  ^a^ Mutually adjusted for group (control vs intervention group), time (baseline vs 12-month follow-up), and the interaction term (group × time).  ^b^ Mutually adjusted for group (control vs intervention group), time (baseline vs 12-month follow-up), the interaction term (group × time), sex, age, and educational attainment.  ^c^ Mutually adjusted for group (control vs intervention group), time (baseline vs 12-month follow-up), the interaction term (group × time), sex, age, educational attainment, and policy awareness among employees. | | | | | | |

**Table S7.** Intervention effect on alcohol use measured using the Alcohol Use Disorder Identification Test (AUDIT) scores as a linear outcome among employees with complete outcome information at baseline (n=2,248). Results from linear mixed model.

|  | Alcohol consumption | | | | | |
| --- | --- | --- | --- | --- | --- | --- |
|  | **Beta^a^** | **95% CI** | **Beta^b^** | **95% CI** | **Beta^c^** | **95% CI** |
| Group, control group=ref. | **-0.51** | -0.78, -0.23 | -0.17 | -0.45, 0.11 | 0.12 | -0.18, 0.42 |
| Time, baseline=ref. | -0.10 | -0.41, 0.21 | -0.02 | -0.32, 0.29 | -0.06 | -0.37, 0.25 |
| Interaction (group×time) | **0.58** | 0.04, 1.13 | 0.27 | -0.26, 0.81 | -0.02 | -0.56, 0.52 |
| CI: Confidence Interval  Bold font: p < 0.05  ^a^ Mutually adjusted for group (control vs. intervention group), time (baseline vs. 12-months follow-up), and the interaction term (group × time)  ^b^ Mutually adjusted for group (control vs. intervention group), time (baseline vs. 12-months follow-up), the interaction term (group × time), sex, age, and educational attainment  ^c^ Mutually adjusted for group (control vs. intervention group), time (baseline vs. 12-months follow-up), the interaction term (group × time), sex, age, educational attainment, and policy awareness among employees | | | | | | |

**Table S8**. The effects of the prevention program on policy awareness among employees in the analytical sample (n=853). Results from multilevel ordered logistic regression.

|  | Supportive organization  to oneself | | Supportive organization to colleagues | | Alcohol policy  knowledge | |
| --- | --- | --- | --- | --- | --- | --- |
|  | **OR^a^** | **95% CI** | **OR^a^** | **95% CI** | **OR^a^** | **95% CI** |
| Group, control group=ref. | **1.82** | 1.75, 1.93 | **1.83** | 1.73, 1.93 | **1.49** | 1.43, 1.56 |
| Time, baseline=ref. | **1.09** | 1.08, 1.10 | **1.07** | 1.07, 1.08 | **1.57** | 1.49, 1.65 |
| Interaction (group × time) | **1.11** | 1.09, 1.12 | **1.08** | 1.07, 1.09 | **0.77** | 0.74, 0.79 |
| CI: Confidence Interval; OR: Odds Ratio  Bold font: p < 0.05  ^a^ Mutually adjusted for group (control vs intervention group), time (baseline vs 12-month follow-up), and the interaction term (group × time). | | | | | | |
